# Occurrence, distribution, and genetic diversity of faba bean viruses in China

**DOI:** 10.3389/fmicb.2024.1424699

**Published:** 2024-06-19

**Authors:** Zongdi Li, Jiachao Qin, Yuxiang Zhu, Mimi Zhou, Na Zhao, Enqiang Zhou, Xuejun Wang, Xin Chen, Xiaoyan Cui

**Affiliations:** ^1^Institute of Industrial Crops, Jiangsu Academy of Agricultural Sciences/Jiangsu Key Laboratory for Horticultural Crop Genetic Improvement, Nanjing, Jiangsu, China; ^2^Department of Economic Crops, Yanjiang Institute of Agricultural Sciences, Jiangsu Academy of Agricultural Sciences, Nantong, Jiangsu, China

**Keywords:** faba bean, viruses, small RNA sequencing, incidence, distribution, genetic diversity, China

## Abstract

With worldwide cultivation, the faba bean (*Vicia faba* L.) stands as one of the most vital cool-season legume crops, serving as a major component of food security. China leads global faba bean production in terms of both total planting area and yield, with major production hubs in Yunnan, Sichuan, Jiangsu, and Gansu provinces. The faba bean viruses have caused serious yield losses in these production areas, but previous researches have not comprehensively investigated this issue. In this study, we collected 287 faba bean samples over three consecutive years from eight provinces/municipalities of China. We employed small RNA sequencing, RT-PCR, DNA sequencing, and phylogenetic analysis to detect the presence of viruses and examine their incidence, distribution, and genetic diversity. We identified a total of nine distinct viruses: bean yellow mosaic virus (BYMV, *Potyvirus*), milk vetch dwarf virus (MDV, *Nanovirus*), vicia cryptic virus (VCV, *Alphapartitivirus*), bean common mosaic virus (BCMV, *Potyvirus*), beet western yellows virus (BWYV, *Polerovirus*), broad bean wilt virus (BBWV, *Fabavirus*), soybean mosaic virus (SMV, *Potyvirus*), pea seed-borne mosaic virus (PSbMV, *Potyvirus*), and cucumber mosaic virus (CMV, *Cucumovirus*). BYMV was the predominant virus found during our sampling, followed by MDV and VCV. This study marks the first reported detection of BCMV in Chinese faba bean fields. Except for several isolates from Gansu and Yunnan provinces, our sequence analysis revealed that the majority of BYMV isolates contain highly conserved nucleotide sequences of coat protein (CP). Amino acid sequence alignment indicates that there is a conserved NAG motif at the N-terminal region of BYMV CP, which is considered important for aphid transmission. Our findings not only highlight the presence and diversity of pathogenic viruses in Chinese faba bean production, but also provide target pathogens for future antiviral resource screening and a basis for antiviral breeding.

## Introduction

Originating from the Mediterranean and Central Asia (Caracuta et al., 2015; Kosterin, 2015; Multari et al., 2015), faba bean (*Vicia faba* L.) is a highly important cool-season legume crop utilized both for human and livestock nutrition (Gu et al., 2020; Meng et al., 2021; Martineau-Côté et al., 2022). Faba bean is cultivated throughout the globe due to its high seed protein content, nitrogen fixation, and good adaptability (Singh et al., 2013; Barbieri et al., 2023). According to the FAO, China has maintained its position as the largest faba bean producer for the last two decades, with 8.11 × 10^5^ hectares of dedicated farmland and 1.69 × 10^6^ metric tons of total yield (dry beans) in 2020, both accounting for more than 30% of the world’s total (FAOSTAT, 2022). In China, faba bean is classified as winter or spring ecotypes based on sowing period (Tian et al., 2007; Wang et al., 2012). The winter ecotype accounts for approximately 85% of the total sown faba bean area in China and is mainly distributed in southern regions, such as Sichuan, Yunnan, Jiangsu, and Hubei provinces (Wang et al., 2012). The spring ecotype is primarily cultivated in northern regions, such as Gansu, Qinghai, Inner Mongolia, and Tibet (Wang et al., 2012).

As faba bean production expands, there has been an increase in the detection of pathogenic viruses (Jones, 2021). Currently, over 50 viruses are known to infect faba bean worldwide (Fresno et al., 1997; Babin et al., 2000; Bellardi and Rubies-Autonell, 2001; Abraham et al., 2010; Nakahara et al., 2011; Makkouk et al., 2012; Kaur et al., 2013; Martin et al., 2014; Worrall et al., 2015; Kraberger et al., 2018; Congdon et al., 2023), more than 20 of which have been reported in China (Meng et al., 2022). These viruses mainly cause symptoms such as chlorosis, etiolation, necrosis, mosaic, mottling, leaf curl, and dwarfism. The yield losses caused by these viruses range from 5% to 20%, up to 90% in severely infected fields (Adhikari et al., 2021; Jones, 2021).

In recent decades, serological and molecular biological technologies have been extensively used to diagnose plant viruses (Rubio et al., 2020; Buja et al., 2021; Mehetre et al., 2021). With the development of gene sequencing technologies, high-throughput sequencing (HTS) has increasingly become an important diagnostics tool, owing to its ability to detect ultra-low levels of viruses and identify novel DNA or RNA viruses (Maree et al., 2018; Villamor et al., 2019; Minicka et al., 2020). Recently, small RNA (sRNA) sequencing has emerged as a highly effective method of diagnosing plant viruses (Pooggin, 2018; Santala and Valkonen, 2018). Also known as gene silencing, RNA interference (RNAi) based antiviral defense is a part of the natural plant immune system (Csorba et al., 2009). During viral replication, plant viruses typically form double-stranded RNA (dsRNA) structures that can be recognized by plants and processed into sRNAs consisting of 21–24 nucleotides (nt) (Tian et al., 2007). Reassembling these sRNAs into longer strands and comparing them with sequences available in public databases enables virus identification (Pooggin, 2018; Santala and Valkonen, 2018).

Effective disease management strategies rely heavily on the detection of viruses and the analysis of their prevalence, distribution, and gene diversity. However, few studies have investigated faba bean viruses in diverse Chinese growing regions. In this study, we comprehensively surveyed faba bean viruses in Chongqing municipality and Jiangsu, Anhui, Yunnan, Guangxi, Sichuan, Gansu, and Hubei provinces. We then employed sRNA sequencing and RT-PCR to study the incidence, distribution, and prevalence of the collected viruses. Furthermore, phylogenetic analysis and sequence demarcation tool (SDT) were utilized to investigate the molecular variability of the predominantly identified virus, bean yellow mosaic virus (BYMV).

## Materials and methods

### Plant material

**FIGURE 1:**
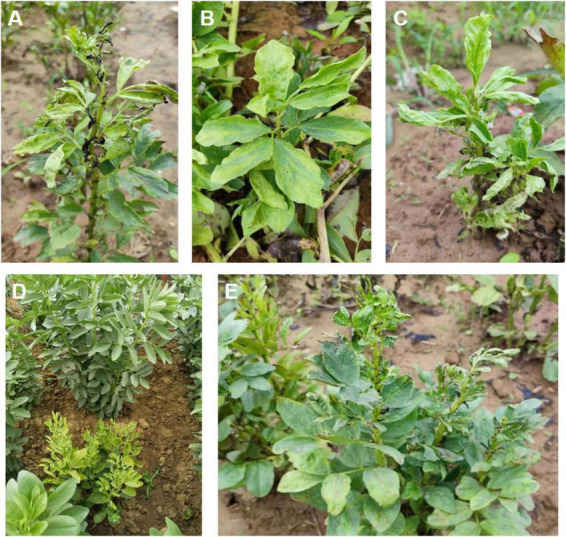
Leaf samples were collected from faba plants displaying major virus-like symptoms. Major symptoms include **(A)** shrinkage and wilting, **(B)** mosaic, **(C)** shrinkage and mosaic, **(D)** dwarfing and chlorosis, and **(E)** chlorosis, leaf curling, and dwarfing.

**FIGURE 2:**
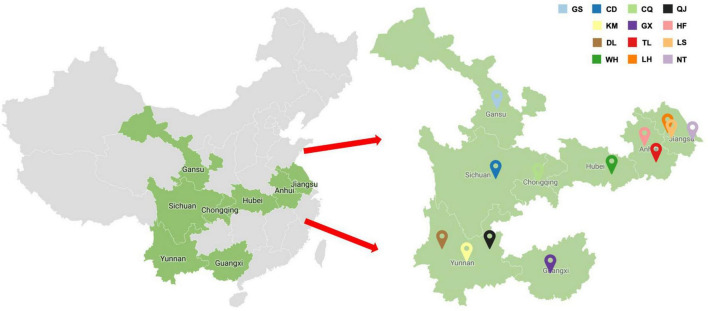
Symptomatic faba bean leaf samples were collected from fields near cities within Jiangsu, Anhui, Gansu, Sichuan, Yunnan, Guangxi, Hubei, and Chongqing (GS, Dingxi; CD, Chengdu; KM, Kunming; DL, Dali; WH, Wuhan; CQ, Chongqing; GX, Nanning; TL, Tongling; LH, Liuhe; QJ, Qujing; HF, Hefei; LS, Lishui; NT, Nantong).

A total of 287 faba bean leaf samples displaying virus-like symptoms were collected from plants cultivated in Jiangsu, Anhui, Yunnan, Guangxi, Sichuan, Gansu, Hubei, and Chongqing over three consecutive years (2019–2021) (Figure 1). The number of samples and the cities nearest to each sample collection site were as follows: 52 from Lishui region, Nanjing city, Jiangsu; 116 from Liuhe region, Nanjing city, Jiangsu; 4 from Nantong city, Jiangsu; 5 from Tongling city, Anhui; 23 from Hefei city, Anhui; 10 from Qujing city, Yunan; 17 from Kunming city, Yunan; 21 from Dali city, Yunnan; 17 from Nanning city, Guangxi; 5 from Chongqing municipality; 7 from Chengdu city, Sichuan; 4 from Dingxi city, Gansu; and 6 from Wuhan city, Hubei (Figure 2 and Supplementary Table 1). Each collected leaf sample was immediately frozen with liquid nitrogen and stored at −80°C.

### RNA extraction and sRNA sequencing

Total RNA was extracted from each sample using the RNAprep Pure Plant Kit (TIANGEN, Beijing, China) according to the manufacturer’s instructions. For sRNA sequencing, libraries were constructed using the TruSeq small RNA library preparation kit (Illumina, San Diego, CA, USA) with total RNA extracted from four separate pooled samples, based on symptom type. The libraries were sequenced on the HiSeq 4000 platform at Lianchuan Biotechnology Co., Ltd. (Hangzhou, China).

### Analysis of sRNA sequencing data

The raw sRNA sequencing data was uploaded to the NCBI database under the accession number PRJNA1092856. Clean reads were obtained by trimming adapter sequences and removing low-quality reads. The reads were then assembled into contigs using Velvet 1.1 and Oases 0.2.07 with *k*-mers parameters of 15 and 17 (Schulz et al., 2012). Sequence alignments were conducted using BLASTn and BLASTx algorithms to search for the resulting contigs in the Genbank viral sequence database.

### Virus detection by RT-PCR

To detect viruses in faba bean samples, cDNA was synthesized from total RNA using a FastKing RT Kit (with gDNase) (TIANGEN, Beijing, China). A mixture of 500 ng RNA template and 2 μl of 5× gDNA buffer was brought to a total volume of 10 μl with RNase-free and then incubated at 42°C for 3 min, followed by 3 min of cooling on ice. Next, 2 μl of 10× Fast RT buffer, 2 μl of FQ-RT Primer Mix, 1 μl of RT Enzyme Mix, and 5 μl of RNase-free water were added to the mixture and the reaction was incubated at 42°C for 15 min and denatured at 95°C for 3 min. Each PCR reaction contained 1 μl of cDNA template, 1 μl of each primer, 12.5 μl of 2× Ftaq PCR MasterMix (ZOMANBIO, Beijing, China), and 9.5 μl of RNase-free water. PCR amplifications were performed using the following program: initial denaturation at 98°C for 3 min; 35 cycles of denaturation at 98°C for 10 s, annealing at 55°C for 15 s, and extension 72°C for 34 s; and a final extension at 72°C for 2 min. Primer sequences designed to detect bean common mosaic virus (BCMV), BYMV, soybean mosaic virus (SMV), pea seed-borne mosaic virus (PSbMV), broad bean wilt virus (BBWV), vicia cryptic virus (VCV), beet western yellows virus (BWYV), cucumber mosaic virus (CMV), and milk vetch dwarf virus (MDV) are listed in Supplementary Table 2. PCR results were visualized by electrophoresis on a 1% agarose gel. PCR products were extracted from the gels with a DNA Gel Extraction Kit (TSINGKE, Beijing, China) following the manufacturer’s instructions. All purified PCR products were subjected to DNA sequencing by Tsingke Biotech Co., Ltd.

### Sequencing analysis

Viral reference sequences of the BYMV coat protein (CP) were acquired from Genbank (Supplementary Table 3). The accession numbers of the BYMV CP sequences generated in this study are shown in Supplementary Table 4. Sequence alignment was conducted with ClustalW software using default parameters. The alignment data was then used to create a phylogenetic tree with MEGA v11.0 software. The parameters were set as follows: statistical method = neighbor-joining; test of phylogeny = bootstrap method (replication: 1,000); substitution type = nucleotide; model/method = maximum composite likelihood; substitution to include = d: transitions + transversions; rates among sites = uniform; pattern among lineages: same (homogeneous); gaps/missing data treatment = pairwise deletion; and number of treads = 7. Sequence alignment was carried out using SDT with the following parameters: alignment programs = muscle and sequence clustering was performed using a neighbor-joining tree. Jalview software was utilized to construct the multiple sequence alignment image.

## Results

### Detection of viruses by sRNA sequencing

The sRNA sequencing results yielded 22,400,291, 21,338,999, 26,794,677, and 26,507,753 clean reads from four pooled samples, with a GC content of approximately 50% (Supplementary Table 5). These resulted in 735, 405, 395, and 410 contigs, with an average length of less than 300 nt (Supplementary Table 5). In the FB-1 sample, three contigs showed 95.9%–100% nt sequence similarities with the BYMV genomic sequence, and 19 contigs showed 96.2%–100% nt sequence similarities with the VCV genomic sequence. In the FB-2 sample, seven contigs showed 98.8%–99.3% nt sequence similarities with the BYMV genomic sequence, and five contigs showed 93.5%–100% nt sequence similarities with the BWYV genomic. In the FB-3 sample, 12 contigs showed 94.4%–99.4% nt sequence similarities with the BYMV genomic sequence, 27 contigs showed 90.1%–100% nt sequence similarities with the MDV genomic sequence, and 10 contigs showed 95.2%–100% nt sequence similarities with the PSbMV genomic sequence. Finally, in the FB-4 sample, 15 contigs showed 98.4%–99.1% nt sequence similarities with the BYMV genomic sequence and 35 contigs showed 90.5%–100% nt sequence similarities with the MDV genomic sequence (Table 1 and Supplementary Table 6).

**TABLE 1:** The number of contigs mapped with known viruses in pooled samples.

Sample	Number of contigs				
	BYMV	VCV	BWYV	MDV	PSbMV
FB-1	3	19	0	0	0
FB-2	7	0	5	0	0
FB-3	12	0	0	27	10
FB-4	15	0	35	0	0

### RT-PCR virus validation

**FIGURE 3:**
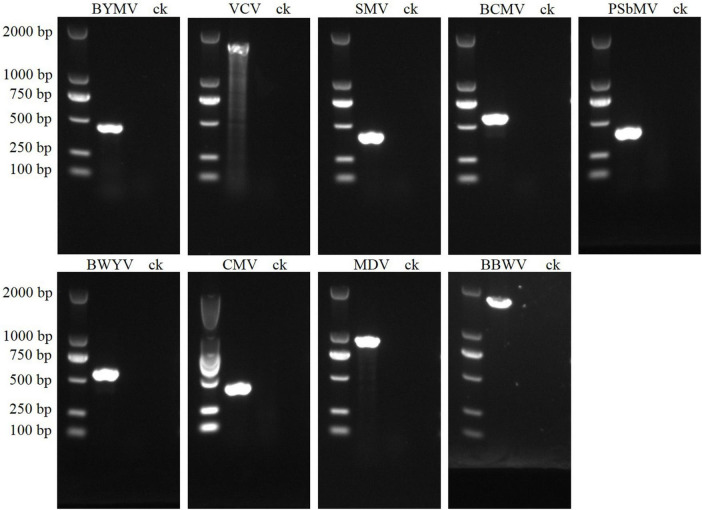
PCR validation for the presence of nine viruses in faba bean leaf samples. The sizes of virus-specific bands were as follows: BYMV, 400 bp; VCV, 1,500 bp; SMV, 400 bp; BCMV, 500 bp; PSbMV, 400 bp; BWYV, 550 bp; CMV, 400 bp; MDV, 900 bp; BBWV, 1,500 bp.

Our sRNA sequencing analysis revealed the presence of BYMV, VCV, BWYV, MDV, and PSbMV in the four pooled leaf samples. Previous researches have indicated that SMV, BCMV, and CMV are commonly detected in faba beans (Fresno et al., 1997; Najar et al., 2003; Golnaraghi et al., 2004; Makkouk et al., 2012), and BBWV was widely distributed in faba bean-growing fields in China (Meng et al., 2022). We designed nine species-specific primer pairs to detect BYMV, VCV, SMV, BCMV, PSbMV, BWYV, CMV, MDV, and BBWV, and our RT-PCR results confirmed the presence of all. In the gel electrophoresis, the PCR products of BYMV, VCV, SMV, BCMV, PSbMV, BWYV, CMV, MDV, and BBWV showed distinct bands of 400, 1,500, 400, 600, 400, 550, 400, 900, and 1,500 bp, respectively (Figure 3). Following purification and Sanger sequencing, a BLASTn algorithm search of the NCBI confirmed the presence of all nine viruses. Since BBWV comprises two strains: BBWV-1 and BBWV-2, the BBWV isolates identified in this study showed 96% nt sequence similarities with BBWV-2.

### Virus prevalence and distribution

Our RT-PCR assays revealed the presence of viruses in 166 of the 287 samples (Table 2). The highest prevalence of viruses was detected in samples collected from Dali (100.0%), followed by Hefei (95.7%), Chengdu (85.7%), Dingxi (75.0%), Nantong (75.0%), and Kunming (58.8%) (Table 2).

**TABLE 2:** Virus incidence in samples collected from different regions.

Region	Number of samples tested	Number of positive samples	Incidence (%)
Lishui	52	16	30.8
Liuhe 1	82	49	59.8
Liuhe 2	34	18	52.9
Nantong	4	3	75.0
Tongling	5	2	40.0
Hefei	23	22	95.7
Qujing	10	4	40.0
Kunming	17	10	58.8
Dali	21	21	100.0
Nanning	17	8	47.1
Chongqing	5	2	40.0
Chengdu	7	6	85.7
Dingxi	4	3	75.0
Wuhan	6	2	33.3
Total	287	166	57.8

**FIGURE 4:**
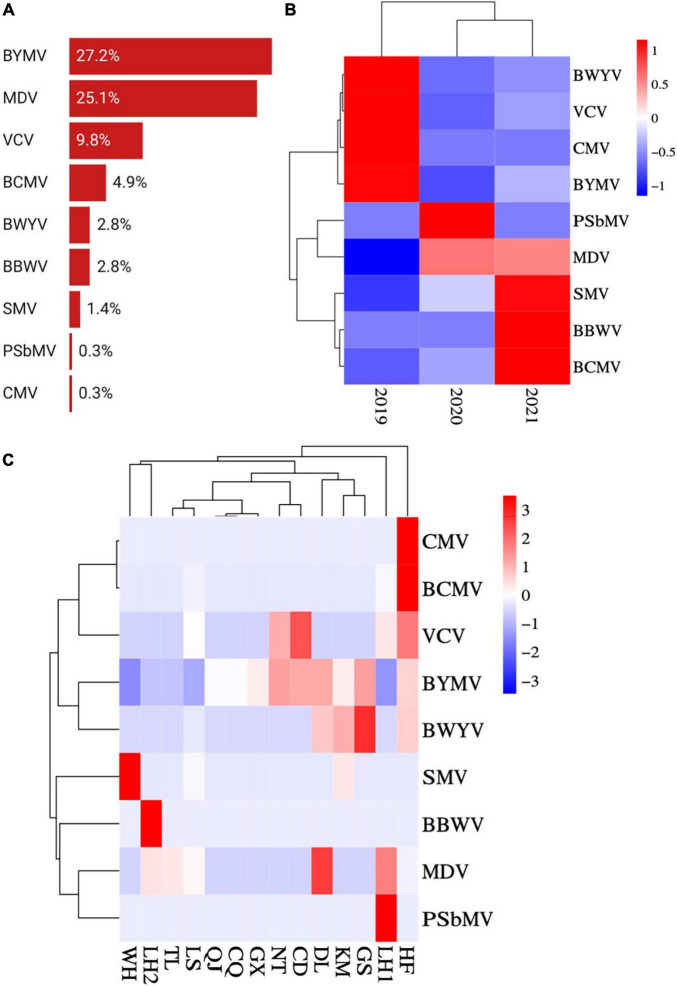
Prevalence and distribution of faba bean viruses in 13 predominant growing regions. **(A)** Incidences (infected samples/total samples) of BYMV, VCV, SMV, BCMV, PSbMV, BWYV, CMV, MDV, and BBWV. **(B)** Heat map of BYMV, VCV, SMV, BCMV, PSbMV, BWYV, CMV, MDV, and BBWV incidences (infected samples/total samples per year) between 2019 and 2021. Red represents a high incidence and blue represents a low one. **(C)** Heat map of BYMV, VCV, SMV, BCMV, PSbMV, BWYV, CMV, MDV, and BBWV incidences (infected samples/total samples per region) between regions (WH, Wuhan; LH, Liuhe; TL, Tongling; LS, Lishui; QJ, Qujing; CQ, Chongqing; GX, Nanning; NT, Nantong; CD, Chengdu; DL, Dali; KM, Kunming; GS, Dingxi; HF, Hefei). Red represents a high incidence and blue represents a low one.

Among the nine virus species, BYMV was detected with the highest incidence (27.2%), followed by MDV (25.1%) and VCV (9.8%) (Figure 4A). SMV, BCMV, PSbMV, BWYV, CMV, and BBWV were detected in fewer than 5% of samples. The incidence of MDV was lower in samples collected in 2019 than in those from 2020 and 2021 (Figure 4B). Conversely, BYMV, VCV, and BWYV were more highly represented in samples collected in 2019 than those from 2020 and 2021. SMV, BCMV, and BBWV were more prevalent in samples collected in 2021 than those from 2019 and 2020 (Figure 4B). Among the top three prevalent viruses, BYMV was detected in samples from all surveyed regions except for Wuhan; MDV was detected in Jiangsu, Anhui, and Yunnan provinces; and VCV was detected in Jiangsu, Anhui, and Sichuan provinces. Additionally, SMV was detected in samples from Jiangsu, Yunnan, and Hubei provinces; BCMV was detected in Jiangsu and Anhui provinces; BWYV was detected in Jiangsu, Anhui, Yunnan, and Gansu provinces; BBWV was only detected in Jiangsu; and PSbMV and CMV were each detected once in samples from Jiangsu and Anhui, respectively (Figure 4C).

### Mixed virus infections

**FIGURE 5:**
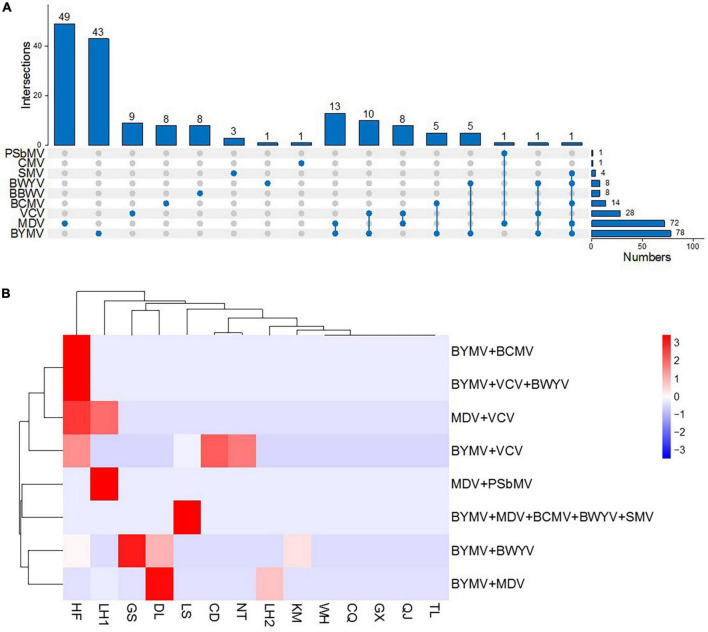
Faba bean samples infected with multiple viruses. **(A)** Intersections of all nine viruses. **(B)** Heat map of mixed virus infections (mixed-infection sample/total samples per region) in various regions (HF, Hefei; LH, Liuhe; GS, Dingxi; DL, Dali; LS, Lishui; CD, Chengdu; NT, Nantong; KM, Kunming; WH, Wuhan; CQ, Chongqing; GX, Nanning; QJ, Qujing; TL, Tongling). Red represents a high incidence and blue represents a low one.

Several of the faba bean plants in our study were infected with two or more viruses at a time (Figure 5A), a phenomenon frequently seen in nature. Among these, the most frequent combination was BYMV and MDV, which was found in 13 samples from Liuhe (5/13) and Dali (8/13). Mixed infections of BYMV and VCV were found in 10 samples from Lishui (2/10), Nantong (1/10), Hefei (5/10), and Chengdu (2/10). Additionally, combinations of VCV and MDV were found in eight samples from Liuhe (6/8) and Hefei (2/8); BYMV and BCMV were found in five samples from Hefei; BYMV and BWYV were found in five samples from Hefei (1/5), Kunming (1/5), Dali (2/5), and Dingxi (1/5); PSbMV and MDV were found in one sample from Liuhe; and BYMV, VCV, and BWYV were found in one sample from Hefei. Notably, one sample from Lishui was found to be infected with five viruses: BYMV, SMV, BCMV, BWYV, and MDV (Figure 5B).

### Genetic diversity of BYMV isolates

Bean yellow mosaic virus represents the most prevalent and widely distributed virus among the nine detected viruses in this study. Seventy-eight samples tested positive for BYMV, accounting for 47.0% of the total number of virus-infected samples. The incidence of BYMV was highest in Dingxi (75%) and Nantong (75%), followed by Chengdu (71.4%), Dali (71.4%), Kunming (47.1%), and Nanning (47.1%). To better understand the distribution of BYMV genotypes, we conducted Sanger-sequencing on the CP genes of isolates originating from different regions. To analyze genetic diversity, we searched Genbank and downloaded the BYMV CP sequences of 3 isolates from lupin, 3 from orchid, 3 from gladiolus, 6 from canna, 26 from faba bean, and 1 complete CP sequence of the clover yellow vein virus (ClYVV, as an out-group) (Supplementary Table 2). These sequences were combined with the 32 complete BYMV CP sequences identified in this study to construct a phylogenetic tree.

**FIGURE 6:**
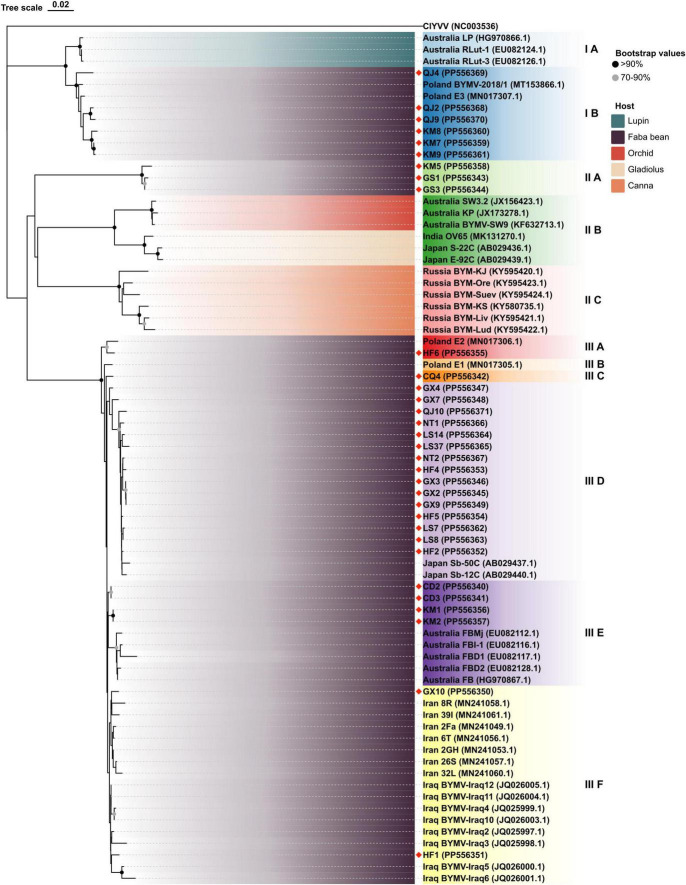
Neighbor-joining phylogenetic tree based on BYMV CP nucleotide sequences obtained from isolates collected from different hosts and regions. Red diamond marks indicate novel sequences obtained in this study.

**FIGURE 7:**
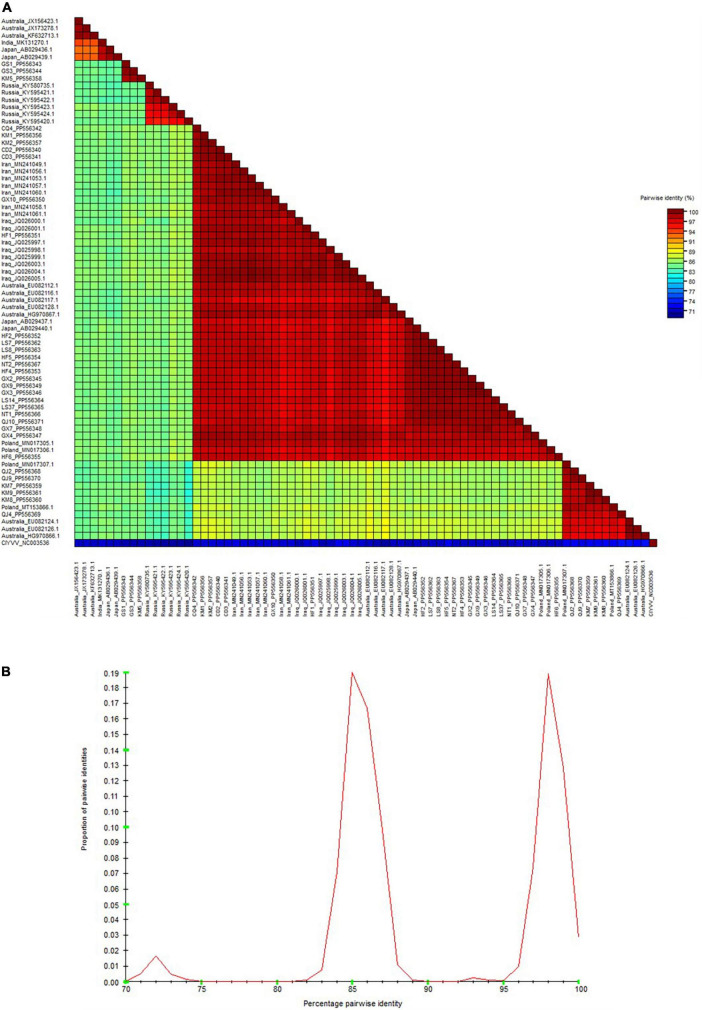
Sequence demarcation tool analysis of BYMV CP sequences. **(A)** Color-coded pairwise identity matrix generated from BYMV CP sequences. Each colored cell represents a percentage identity score between two sequences **(B)** Pairwise identity frequency distribution plot of BYMV CP sequences.

**FIGURE 8:**
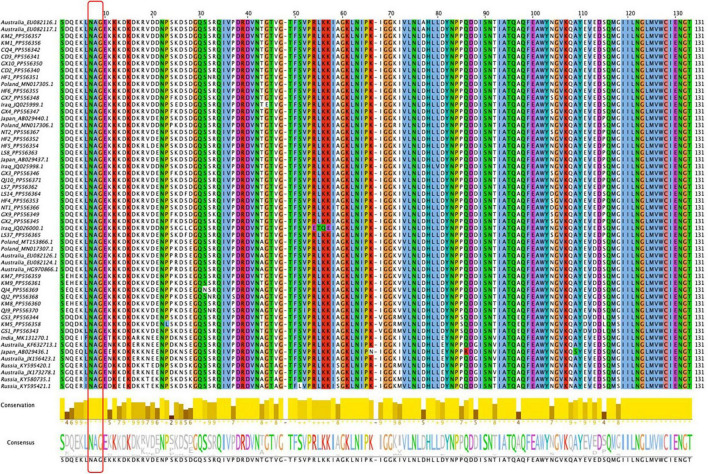
Amino acid sequence comparison of the BYMV CP N-terminal. Sequence alignment was achieved using the MUSCLE method by Jalview software. Conserved amino acids are highlighted with different colors according to the ClustaLx code. The red box signifies the NAG motif.

As depicted by the phylogenetic tree, the 73 total BYMV CP sequences fell into three major groups (Figure 6). Group I A consisted of three isolates from lupin (LP, RLut-1, and RLut-2), and Group I B comprised three isolates from Qujing (QJ2, QJ4, and QJ9) and three isolates from Kunming (KM7, KM8, and KM9). These two groups were most closely related to two faba bean isolates from Poland (BYMV-2018/1 and E3). Group II was divided into three subgroups: Group II A, which contained KM5 and two closely related isolates from Dingxi (GS1 and GS2); Group II B, which encompassed isolates SW3.2, KP, and SW9 from orchid and three closely related isolates from gladiolus (OV65, S-22C, and E-92C); and Group II C, which included six Russian isolates from canna. Group III exclusively comprised isolates from faba bean plants, further divided as follows: Group III A comprised one isolate from Hefei (HF6), and the closely related E2 isolate from Poland; outgroups III B and III C held another Polish isolate (E1) and CQ4 from Chongqing; Group III D contained 15 isolates, GX4, GX7, QJ10, NT1, LS14, LS37, NT2, HF4, GX3, GX2, GX9, HF5, LS7, LS8, and HF2, which were most closely related to 2 isolates from Japan (Sb-50C and Sb-12C); Group III E encompassed 2 isolates from Chengdu (CD2 and CD3), 2 isolates from Kunming (KM1 and KM2), and 5 closely related isolates from Australia (FBMj, FBI-1, FBD1, FBD2, and FB); and Group III F contained all isolates from Iran and Iraq, which were closely related to isolates GX10 from Nanning and HF1 from Hefei (Figure 6). Moreover, we also constructed a phylogenetic tree based on the amino acid sequences of BYMV CP. As is shown in Supplementary Figure 1, the 73 total BYMV CP sequences fell into three major groups: Group I consisted of seven isolates from Iran; Group II comprised one isolate from Australia (FBMj) and one isolate from Iraq (BYMV-Iraq6); Group III exclusively comprised 32 isolates identified in this study. SDT analysis was also applied to study the genetic diversity of BYMV isolates. The results revealed that BYMV isolates collected from the same host exhibited highly conserved nucleotide sequences, while significant variations were observed between isolates from different hosts (Figure 7). Nevertheless, there was significant variation between isolates GS1, GS3, and KM5 with other faba bean isolates. Additionally, several isolates from Qujing (QJ2, QJ4, and QJ9) and Kunming (KM7, KM8, and KM9) showed a high degree of similarity with isolates from lupin (LP, RLut-1, and RLut-3) (Figure 7). The potyvirus CP is a multitasking protein (Martínez-Turiño and García, 2020; Yang et al., 2021), and previous studies have revealed that its N-terminal is indispensable for certain aspects of the viral life cycle, such as virion assembly, virus transmission, virus movement, host adaptation, resistance response, and post-translational modifications (Martínez-Turiño and García, 2020; Yang et al., 2021). To study the conserved motifs of the N-terminal region, we aligned the amino acid sequences of our BYMV CP isolates. The N-terminal region across various isolates was found to be highly conserved (Figure 8). It has been reported that the DAG motif in the N-terminal region of potyvirus CP is critical for aphid transmission (López-Moya et al., 1999; Gadhave et al., 2020). However, a recent study indicated that the CP sequence of BYMV instead contained a NAG motif (Robertson and Coyne, 2009), which was consistent with our results (Figure 8). The NAG motif present at the 7–9 positions of the deduced amino acid residues of CP was highly conserved in all BYMV isolates, regardless of host or region.

## Discussion

This comprehensive survey of faba bean viruses analyzed samples collected from eight provinces/municipalities of China. We detected nine viruses, either as single or combined infections, from sample leaf tissue, including BYMV, VCV, SMV, BCMV, PSbMV, BWYV, CMV, MDV, and BBWV. In our investigation, BYMV, MDV, and VCV ranked as the top three most prevalent viruses in surveyed regions (Figure 4A). BYMV usually causes symptoms on leaves, thereby, it would not have huge impact on the yield if the infection occurring in the middle or late stage of faba bean growth (Radwan et al., 2008). The infections of MDV and VCV on faba bean were found in recent years, which have the tendency to spread in different hosts such as Fabaceae family, garlic, tobacco plant (*Nicotiana tabacum* L.) in China (Zhang et al., 2017, 2020b). The infection of MDV should be detected at the early stage, because it can lead to serious yield losses (Sun et al., 2022). VCV is difficult to be found due to its cryptic symptoms (Blawid et al., 2007). The single infection of VCV is not a big concern to the faba bean production, while it is very likely to cause mixed infections with other viruses, which needs more attention (Blawid et al., 2007).

Previous reports have documented more than 20 viruses infecting faba bean plants in China (Kumari and Makkouk, 2007), including BBWV in Sichuan, Hubei, Zhejiang, Jiangsu, and Yunnan; TuMV in Zhejiang and Jiangsu; SMV in Jiangsu; BBSV in Sichuan, Hubei, Zhejiang, and Jiangsu; BYMV in Jiangsu and Yunnan; BLRV in Hubei and Jiangsu; VCV in Jiangsu; VCV-M in Jiangsu; MDV in Jiangsu and Yunnan; ClYVV in Jiangsu; PSbMV in Yunnan; BBTMV in Anhui and Yunnan; CMV in Jiangsu and Yunnan; BWYV in Yunnan; and BBMV in Yunnan; TMV in Zhejiang (Ding and Li, 1985; Xu et al., 1985, 1989; Zhou et al., 2000; Bao et al., 2007; Kumari et al., 2010; Zhang et al., 2017, 2020a; Meng et al., 2022). Of these, BBWV was reportedly one of the most prevalent (Xu et al., 1985; Meng et al., 2022). However, our investigation only detected BBWV in eight samples from Liuhe, indicating that BBWV was not the major cause of faba bean viral diseases between 2019 and 2021 in most surveyed regions. This study reports the first known occurrences of BYMV in Hefei, Guangxi, Sichuan, Gansu, and Chongqing; VCV in Anhui and Sichuan; BWYV in Jiangsu, Anhui, and Gansu; and MDV in Anhui. BCMV is recognized as one of the most common and destructive viruses of beans (*Phaseolus vulgaris* L.) and various other legumes (Worrall et al., 2015), yet our findings offer the first documented infection of BCMV on faba bean in China. There was a low incidence of this virus in surveyed regions, and the majority of isolates were detected in samples from Hefei (11/14).

Few studies have analyzed mixed viral infections on faba beans (Kumari and Makkouk, 2007), however, our study identified several combinations. In total, 42 samples were found to be infected with two or more virus species. The most prevalent combination was BYMV and MDV (13/42), followed by BYMV and VCV (10/42), and MDV and VCV (8/42). Notably, one sample was infected with three viruses—BYMV, VCV, and BWYV and one sample was infected with five viruses—BYMV, MDV, BCMV, BWYV, and SMV. BYMV was detected in six out of eight virus combinations, suggesting that it was more widespread. The viruses documented in these combinations induce similar mosaic symptoms, making it difficult to visually distinguish them. Therefore, further diagnostic methods should be developed to more accurately study these virus combinations.

During this investigation, BYMV was the most commonly detected virus. The virus is distributed worldwide and can cause up to 80% yield losses in faba bean production (Kumari and Makkouk, 2007). BYMV infects a wide range of legume crops and can also invade certain flowering plants such as gladiolus, orchid, and clover, which serve as alternative hosts (Kumari and Makkouk, 2007). Our phylogenetic analysis of BYMV CP sequences demonstrated that the isolates identified in this study, together with isolates from previous research, could be distributed into three groups, with placement largely dependent on the host plant (Figure 6). Out of the 32 BYMV CP sequences obtained from this study, 23 were placed in Group III, all of which were isolated from faba bean. Iran and Iraq are generally regarded as the origin of the faba bean, however, only two isolates identified in this study, GX10 and HF1, were closely related to representatives from these countries (Figure 6). This group also contained six isolates from Yunnan province and two isolates from Poland. These were closely related to three isolates from lupin but were distant from other BYMV isolates from faba bean (Figure 6). Notably, isolates QJ2, Qj4, and QJ9 from Qujing and isolates KM7, KM8, and KM9 from Kunming were closely related to isolates from lupin, suggesting they are likely transmitted from lupin to faba bean by aphids. Therefore, it may be beneficial to plant faba bean plants away from other legume crops that are susceptible to BYMV. Our phylogenetic tree illustrates that of 15 isolates in Group III E, LS7, LS8, LS14, LS37, NT1, and NT2 were from Jiangsu. These six isolates were found to be closely related to two isolates from Japan, Sb-12C and Sb-50C (Figure 6). Previous studies have demonstrated that BYMV could be transmitted through seeds (Dell’Olmo et al., 2023), suggesting that the high degree of homogeneity between Jiangsu and Japanese isolates may be due to the increasingly active seed trade between the two regions. Therefore, effective disease management is dependent on the use of virus-free seeds. Increasingly popular with the International Committee on Taxonomy of Viruses (ICTV), SDT is a virus classification tool based on pairwise sequence alignment and identity calculation (Muhire et al., 2014). Our research employed this method to analyze the nucleotide sequences of BYMV CP extracted from individual isolates. This analysis further confirmed our phylogenetic tree, indicating that most faba bean isolates contained highly conserved nucleotide sequences, except for several isolates from Yunnan and Gansu (Figure 7A), which could evolve into distinct strains with unique virulence. A sequence alignment also revealed highly conserved motifs of the N-terminal of BYMV CPs (Figure 8). The NAG motif, associated with aphid transmission, was highly conserved in all BYMV isolates. Previous studies have reported that although the amino acid following the D(N)AG motif was not highly conserved in potyviruses, the local presence of an acidic Glu or Asp residue in the tobacco vein mottling virus (TVMV, *Potyvirus*) CP markedly reduces aphid transmissibility (Atreya et al., 1995). However, our study identified a Glu following the NAG motif that was highly conserved across BYMV isolates (Figure 8). Since the analyzed BYMV isolates were all collected from production fields, these viruses were likely transmitted by vectors. Therefore, we posit that this Glu likely does not affect BYMV aphid transmissibility, but further assays should be conducted to confirm this hypothesis.

In this study, we comprehensively investigated faba bean viruses in the production fields of eight provinces/municipalities of China and detected nine distinct viruses. The presence of these viruses was verified by sRNA sequencing and RT-PCR assays. Finally, phylogenetic, SDT analyses, and sequence alignments were conducted to explore the genetic diversity of BYMV, which was the predominantly detected virus. Taken together, this report offers insights into viral threats to Chinese faba bean production, offering guidance for future antiviral breeding and disease management.

## Data availability statement

The datasets presented in this study can be found in online repositories. The names of the repository/repositories and accession number(s) can be found in the article/Supplementary material.

## Author contributions

ZL: Data curation, Formal analysis, Investigation, Methodology, Writing – original draft. JQ: Data curation, Formal analysis, Investigation, Methodology, Writing – original draft. YZ: Investigation, Methodology, Writing – original draft. MZ: Investigation, Methodology, Writing – original draft. NZ: Resources, Software, Writing – original draft. EZ: Resources, Software, Writing – original draft. XW: Funding acquisition, Project administration, Writing – review & editing. XC: Funding acquisition, Project administration, Supervision, Writing – review & editing. XYC: Funding acquisition, Project administration, Supervision, Writing – original draft, Writing – review & editing.
