# Supplementary figures and images for: Occurrence, distribution, and genetic diversity of faba bean viruses in China
Source: Front Microbiol. 2024 Jun 19;15:1424699. doi: 10.3389/fmicb.2024.1424699 (PMC11219563; doi:10.3389/fmicb.2024.1424699)

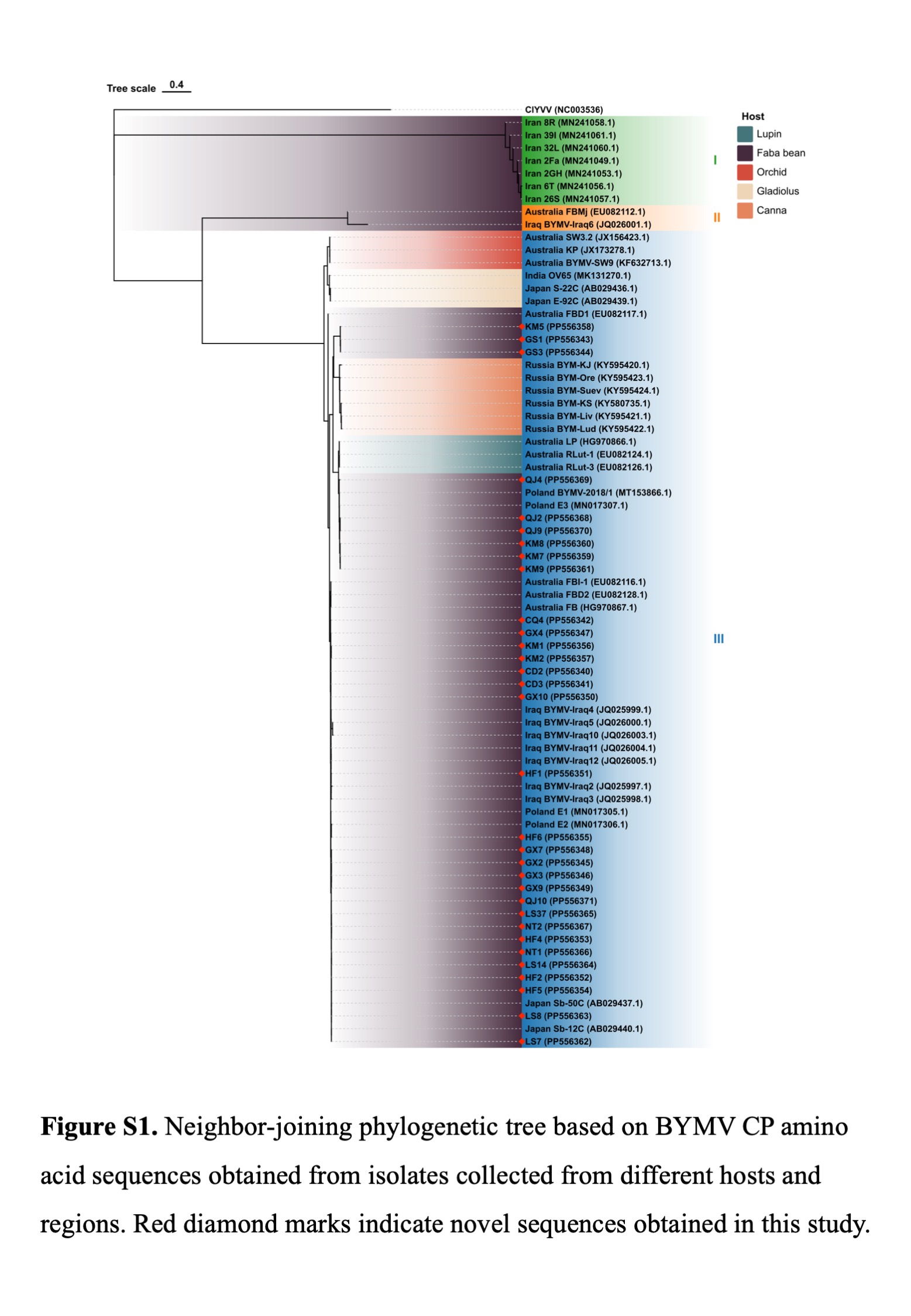

Supplement: Supplementary file 2 [file Image_1.JPEG]
